# Correlations of knowledge and preference of medical students for a specialty career: a case-study of youth health care

**DOI:** 10.1186/1471-2458-8-14

**Published:** 2008-01-14

**Authors:** Marc BM Soethout, Olle TJ ten Cate, Gerrit van der Wal

**Affiliations:** 1 VU University Medical Center, Department of Public and Occupational Health, EMGO Institute, Amsterdam, The Netherlands; 2 University Medical Center Utrecht, School of Medical Sciences, Utrecht, The Netherlands

## Abstract

**Background:**

Medical students develop interest in a specialty career during medical school based on knowledge and clinical experience of different specialties. How valid this knowledge is and how this knowledge relates to the development of preference for a specialty is not known. We studied their "subjective" knowledge of a specialty (students' reported knowledge) with "objective" knowledge of it (students actual knowledge as compared to reports of specialists) and their preference for this specialty at different stages of education, and used youth health care as a case study.

**Methods:**

Students from all years in two medical schools (N = 2928) were asked to complete a written questionnaire including (a) a statement of their knowledge of youth health care (YHC) ("subjective knowledge"), (b) their preference for a YHC career and (c) a list of 47 characteristics of medical practice with the request to rate their applicability to YHC. A second questionnaire containing the same 47 characteristics were presented to 20 practicing youth health physicians with the request to rate the applicability to their own profession. This profile was compared to the profiles generated by individual student's answers, resulting in what we called "objective knowledge."

**Results:**

Correlation studies showed that "subjective knowledge" was not related to "objective knowledge" of the YHC profession (r = 0.05), but significantly to career preference for this field (r = 0.29, P < 0.01). Preference for a YHC career hardly correlated with objective knowledge about this profession (r = 0.11, P < 0.05). Students with YHC clerkships showed no better "objective knowledge" about the profession than students without such experience.

**Conclusion:**

Career preference aren't always related to prior experiences, or to actual knowledge of the area. This study shows how careful we should be to trust students' opinions and preferences about specialties; they probably need much guidance in career choice.

## Background

During medical school medical students develop interest in one or more specialty careers based on knowledge and clinical experience of different specialties [1]. It may be assumed that gradually this knowledge of specialties increases, especially by experience during the clerkships. However, we do not know how this knowledge is related to the development of a preference for a specialty. Previous research has shown that the preference for youth health care, one of the main public health specialties in the Netherlands, decrease during medical school, and we used this specialty for a case study [2,3].

Youth health physicians are employed by municipal health services and home care institutions, and they carry out a standardized health care procedure that has been established for all children between 0 and 19 years of age, consisting of regular screening activities (i.e. hearing and visus screening, and vaccination), monitoring of growth and development and the prevention of threats to well-being. They invite the youth (and their parents) for regular check-ups and make referrals to other medical specialties if medical treatment appears to be needed. The prevention of restrictions and problems in the growth and development of youth is therefore their main task in daily practice [4]. This is why they differ from paediatric physicians, who work in hospitals, and mainly treat patients with a disease, who are referred to them by general practitioners or youth health physicians. Youth health care is rather popular among female doctors, and also among first-year medical students. However, during medical school interest in this profession decreases markedly, and it becomes one of the least popular medical careers by the time the students graduate [3,5]. We have the impression that medical students have a very limited perception of youth health care and have very little basis on which to consider a career in this direction. To confirm this impression, we conducted a survey among medical students at the VU University medical center in Amsterdam (VUmc) and the University Medical Center Utrecht (UMCU) in the Netherlands. We investigated medical students' subjective knowledge (students' reported knowledge) of the profession of youth health care with objective knowledge of it (students actual knowledge) as compared to reports of youth health physicians) and preference for this specialty at different stages of education.

The following research questions were addressed:

1. How much do medical students think they know about the specialty of youth health physician (''subjective knowledge")? How much do medical students really know the specialty of youth health physician compared to practicing youth health physicians themselves (''objective knowledge")?

2. Are subjective knowledge, objective knowledge and preference for a specialty career correlated?

We expected an increasingly objective knowledge of the profession of youth health physician during medical school, and especially for those students who get experience in a youth health clerkship. We also expected to find an increase in the number of students with a high subjective knowledge about the profession, especially among those with a preference for a career as a youth health physician or with experience in a youth health clerkship.

## Methods

In September 2002, all 2928 medical students at the VUmc and the UMCU, received a written questionnaire. Students in the first four study years received the questionnaire during a tutorial at the start of the academic year, and completed the questionnaire immediately. Students in their clerkships (final two years) received the questionnaire halfway through and at the end of their clerkships. Students halfway through their clerkships participated in clerkships in different hospitals, so they received the questionnaire by mail, and three reminders were sent. All final-year VUmc students had participated in a two-week public health clerkship, most of them in occupational health care, and a small group in youth health care. In September 2005, all 20 practicing youth health physicians collaborating with both medical schools were sent a similar questionnaire by mail, followed by three reminders. Consent for this study was obtained from all participants when the questionnaires were completed.

The primary question in the student questionnaire was: ''How much would you like to practice in the future as a physician in (name of specialty)?". They were asked to indicate how strong their preference was for a career for each of 37 medical specialties registered in the Netherlands on a 5-point Likert scale (1 = absolutely not, 2 = preferably not, 3 = neutral, 4 = possibly, or 5 = definitively). They were also asked: ''How much do you know about the specialty of (name of specialty)?" to assess their perceived knowledge (subjective knowledge) about these specialties on a 5-point Likert scale (1 = very little, 2 = a little, 3 = moderate, 4 = good or 5 = very good). Finally, questions were asked about 47 general characteristics of the medical profession, phrased as "To what extent does this characteristic apply to the profession of youth health physician?" with a 5-point rating scale, ranging from 1 (very little) to 5 (very much), based on earlier research about student's perception of the medical profession [6]. The youth health physicians were asked to indicate how much these characteristics applied to their daily practice, also on a 5-point scale, ranging from 1 (very little) to 5 (very much). For the analysis the study population was divided into a group of students with a high and low preference for a career as a youth health physician (score of 1 and 5, respectively), a group of students who had high subjective knowledge and low subjective knowledge (score of 5 and 1, respectively) about the profession, a group of final-year students with and without youth health clerkship experience, and a group of practising youth health physicians.

The youth health physicians' answers were recalculated to establish a Youth Health Physician profile (YHP profile). To this end, characteristics with the best internal agreement according to the YHP respondents (standard deviation lower than or equal to 0.80; n = 21) were included in this YHP profile. Characteristics with less agreement (standard deviation larger than 0.80, n = 26) were excluded, because they apparently represent less consensus of opinion within the profession.

Subsequently, differences between the YHP profile characteristics and the corresponding students' scores were calculated, by subtracting the mean score of each of the YHP profile characteristics from the corresponding score of each student. Positive deviations signify an average belief among the students that the importance of a characteristic is greater than it actually is; negative deviations show how students under-value a characteristic. Deviations can have a maximum value of 4 points (when a characteristic is scored 1 by all youth health physicians and 5 by all students, or vice versa). A resulting aggregated total score of deviations of items from the YHP-profile (n = 21) was labelled 'objective knowledge of the profession', (the smaller the deviation, the higher the score for objective knowledge) in contrast with the 'subjective knowledge of the profession' as derived from the questionnaire.

A standardized difference was calculated for all the YHP profile characteristics (n = 21) and the corresponding students [7]. The differences were analysed with Chi-square tests and T-tests, and the correlation between the characteristics of the profession (YHP profile), subjective and objective knowledge, and preference for a career as a youth health physician were calculated with Spearman's rank correlation coefficients. Where 21 multiple parallel significance test were executed, we adapted the significance level to avoid alpha errors; we considered P < (0.05/21 = 0.002, i.e. P < 0.001 to reflect significant differences.

## Results

All 2928 medical students at the VUmc and the UMCU received a questionnaire; 2342 questionnaires were returned (80%). The response rate for the practicing youth health physicians (N = 20) was 100%. The response rate for the students differed between the two medical schools (VUMC 93%, UMCU 67%), due to a difference in the method of distributing the questionnaires. The difference in gender between the respondents from the two medical centers was much the same (66% women and 34% men), and in line with the general medical student population.

### Preference and objective knowledge

Of all students (n = 149), 7% had a high preference for a career in youth health care. Most of them were in their first three study years, and only 10 of them were in their last two study years. Of the first-year students, 11% had a high preference and 6% had a low preference for a career in youth health care. These percentages were 1% and 24%, respectively, in the final year of medical school (p = 0.000).

**Figure 1:**
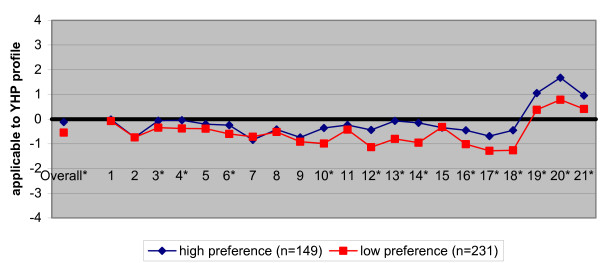
Characteristics of the medical profession, considered applicable to youth health (YHP profile) by students with a high preference and students with a low preference for a career as a youth health physician. Students' views were compared with the standardized YHP profile as reported by professionals (the zero-line) (*p < 0.001)

Figure 1 shows that students with a high preference for a career as a youth health physician do differ significantly overall in their objective knowledge of the profession from those with a low preference (p < 0.001). The items that were over-valued and under-valued were similar in both groups, with only slight differences in the extent of over and under-valuation. In general, compared to students with a low preference for a career as a youth health physician, the students with a high preference, with regard to the characteristics of the youth health care profession, were more similar to those of the practicing youth health physicians (Table 1).

**Table 1:** Practicing youth health physicians' and medical students' opinion about the applicability of general characteristics of future medical practice to the profession of youth health physician (mean scores).

	Youth health physicians (N = 20)		All students (N = 2342)	
	Mean	Std. Deviation*	Mean	Std. Deviation
1. Young patients (0 – 18 years)	5.0	0.0	4.9	0.3
2. Part-time work possibilities	5.0	0.2	4.2	0.9
3. Advise	4.9	0.4	4.6	0.6
4. Communication skills	4.9	0.4	4.6	0.6
5. Knowledge of psychosocial background	4.9	0.4	4.6	0.7
6. Talk at work	4.9	0.4	4.4	0.8
7. Healthy people	4.8	0.4	4.1	1.1
8. Prevention	4.8	0.4	4.3	0.9
9. Psychosocial complaints	4.7	0.6	3.8	1.0
10. Trust relation with patients	4.6	0.8	3.8	1.2
11. Refer	4.5	0.8	4.2	0.9
12. Knowledge of pathology	4.4	0.6	3.6	1.0
13. Reasoning skills	4.4	0.7	3.9	0.9
14. Improvisation skills	4.3	0.6	3.7	1.1
15. Knowledge of health systems	4.3	0.8	3.9	0.9
16. Thinking at work	4.2	0.8	3.4	1.0
17. Knowledge of science	3.9	0.8	2.9	1.1
18. Stressful work	3.5	0.8	2.7	1.0
19. Knowledge of pharmacotherapy	2.6	0.8	3.2	1.1
20. Treatment of patients	1.9	0.8	3.1	1.3
21. Terminal complaints	1.3	0.6	2.0	1.0
22. Teamwork	4.3	1.0	3.4	1.1
23. Knowledge of epidemiology	4.1	1.2	4.2	0.8
24. Many consults	4.1	1.1	3.6	1.1
25. Simple complaints	3.9	1.2	3.8	0.9
26. Knowledge of anatomy	3.8	0.9	3.3	1.0
27. Consultation	3.7	1.3	3.6	1.0
28. Diversity of work	3.7	0.9	3.2	1.1
29. Diagnostic skills	3.5	0.9	3.5	1.1
30. Practical skills	3.4	1.1	2.7	1.0
31. Routine work	3.4	0.9	4.0	1.0
32. Knowledge of chemistry and physics	3.2	1.2	3.2	1.0
33. Long relation with patients	3.2	1.3	3.0	1.2
34. Chronic complaints	3.1	1.0	3.1	1.2
35. Multiple complaints	3.1	1.2	2.9	1.2
36. Long working days	2.9	1.1	2.8	1.1
37. High income	2.5	1.0	2.9	0.9
38. Practical skills with equipment	2.5	1.1	2.7	1.0
39. Older patients (over 65 years)	2.4	1.8	1.2	0.6
40. Physical work	2.4	1.1	2.7	1.0
41. Technical precise work	2.4	0.9	2.4	1.0
42. Visible results	2.4	0.9	3.0	1.1
43. Guidance of illness	2.3	1.1	3.2	1.3
44. High prestige	2.3	1.0	2.7	1.0
45. Acute complaints	2.2	0.9	2.6	1.3
46. Pain release	1.8	0.9	2.9	1.3
47. Irregular work	1.4	0.9	2.5	1.1

*Nrs.1-21 have sufficient low variance (SD ≤ 0.80 among YH physicians) to be included in a YHP profile. For all YHP profile characteristics and corresponding student's perceived characteristics we calculated a standardized difference of 1.1.

### Objective and subjective knowledge

Only 4% (n = 93) of all students indicated a high level of subjective knowledge about the profession of youth health physician. This percentage was much higher among students with a clerkship experience in youth health care (32%), and among those who had a high preference for a career as a youth health physician (20%).

**Figure 2:**
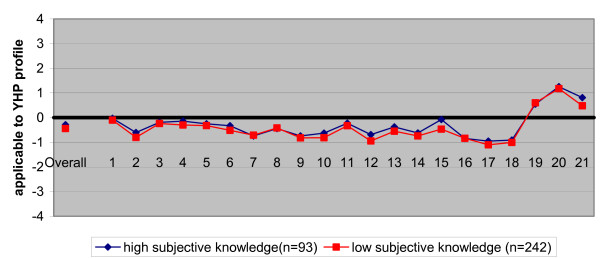
Characteristics of the medical profession, considered applicable to youth health (YHP profile) by students with high subjective knowledge and students with low subjective knowledge about a youth health physician. Students' views were compared with the standardized YHP profile as reported by professionals (the zero-line)

Figure 2 shows a striking resemblance of the student's objective knowledge of the profession between those who had high subjective knowledge and those who had low subjective knowledge. In other words, the amount of knowledge student said they had about the profession had no relationship with the reality at all.

### Objective knowledge and stage of training

**Figure 3:**
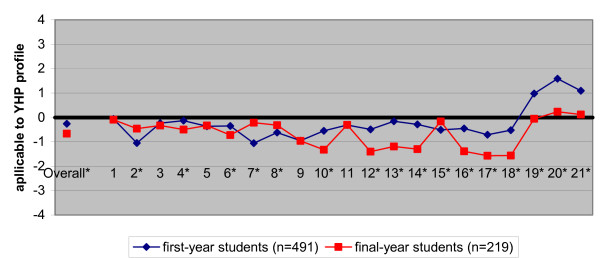
Characteristics of the medical profession, considered applicable to youth health (YHP profile) by first-year students and final-year students. Students' views were compared with the standardized YHP profile as reported by professionals (the zero-line) (*p < 0.001)

Figure 3 shows the difference between the objective knowledge of first-year students and final-year students (students with a youth health clerkship experience were excluded) about the specialty. In general, the first-year students had a significantly higher objective knowledge of the profession (YPH profile) than the final-year students. First-year students had for 9 of the 21 items on average a significantly closer match with the professional reference group, whereas the final-year students had this closer match for only 6 of these items; 6 items showed no difference. The results remained the same when students with experience in a youth health clerkship were included in the group of final-year students (not shown in table).

### Objective knowledge and youth health care clerkship experience

**Figure 4:**
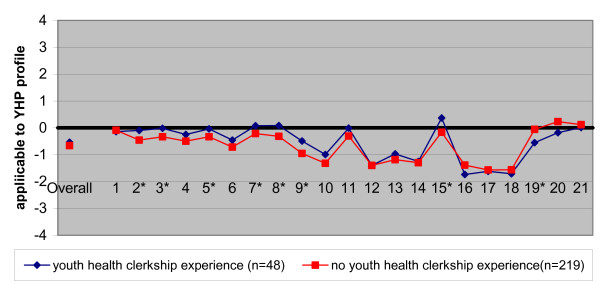
Characteristics of the medical profession, considered applicable to youth health (YHP profile) by students with experience in a youth health clerkship and all other final-year students with clerkship experience. Students' views were compared with the standardized YHP profile as reported by professionals (the zero-line) (*p < 0.001)

Figure 4 shows the objective knowledge of only those students who had completed their clerkships in the final-year of medical school. Again, a striking resemblance was found between the students with and without experience in a youth health care clerkship. Apparently, experience in the field had little effect on student's knowledge of the profession, or on deviations in this knowledge from daily practice.

### Correlation of career preference with subjective and objective knowledge about the profession

As can be expected from the previous findings there was virtually no correlation between 'subjective' and 'objective' knowledge. In addition, we found a very small, but significant, negative correlation between objective knowledge and career preference, indicating that those with a higher preference had a slightly less realistic perception of the profession. Interestingly, however, subjective knowledge of the profession correlated significantly with career preference (r = 0.29). In other words, those who stated that they had knowledge about the profession, even if this was not true, more often had a preference for a career in youth health care (Table 2).

**Table 2:** Correlation of subjective knowledge about the profession of youth health physician, preference for a career as a youth health physician, and objective knowledge of characteristics of a youth health physician by students (* p < 0.05, **p < 0.01)

	Subjective knowledge about YHP	Career preference for YHP	Objective knowledge about YHP
Subjective knowledge about YHP	1.00	0.29*	-0.05**
Career preference for YHP	0.29*	1.00	-0.11*
Objective knowledge about YHP	-0.05**	-0.11*	1.00

## Discussion

Approximately 7% of all medical students have a high preference for a career as a youth health physician, and 4% of all medical students have high subjective knowledge about the profession. These percentages are higher in the first year of medical school than in the final year. Objective knowledge of the profession appears to be influenced very little by preference for a career in the field, and virtually not at all by subjective knowledge of the profession or by specific clerkship experience in the field.

We expected to find that experience in a youth health clerkship would increase the objective knowledge of the YHP. Interestingly, we found almost no correspondence for most aspects of the YHP profile aspects. Students under-value most of the characteristics of the YHP profile, maybe because they only spend two weeks in a youth health clerkship, and that might be too short to be confronted with all these characteristics. The younger students, in particular, may confuse youth health care with paediatrics. We asked students how applicable general characteristics of the profession were to practising youth health physicians. The extent to which these characteristics are also applicable to other specialties is not known, in other words some individual characteristics of the YHP profile, such as ''treatment of patients" are surely also a part of other specialty profiles.

We asked medical students what they knew about medical specialties. Although self-reported knowledge differs from assessed knowledge, it has been reported to have high validity [8]. This is contrary to what we found and this study shows how careful we should be to trust students opinions and preference about specialties; they probably need much guidance in career choice. On the other hand, self-reported knowledge is probably influenced by a student's frame of reference. For instance, many students have met with youth health physicians during primary school. However, during medical school they are introduced to many other specialties that they were not familiar with, and actual experience in youth health care mainly takes place during the clerkships. This might be an explanation for the high percentage of students who think that they have good knowledge about youth health care in their first year of medical school, and a lower percentage over the years. A lower level of knowledge about the profession over the years might also explain the negative correlation between a high preference for a career in youth health care and knowledge about the YHP profile.

An increase in the attention that is paid to public health care in medical schools has resulted in an increase in knowledge and preference for a career in this field [9,10]. One way in which this can be encouraged might be by planning earlier contacts with youth health physicians in the medical curriculum, but clearly experience that is gained in a clerkship does not help much in this respect. Finally, a limitation is that youth health care may differ from large specialties, such as internal medicine or surgery and it would be worthwhile to extend our study to other medical domains.

## Conclusion

Career preference aren't always related to prior experiences, or to actual knowledge of the area. This study shows how careful we should be to trust students' opinions and preferences about specialties; they probably need much guidance in career choice.

## Competing interests

The author(s) declare that they have no competing interests.

## Authors' contributions

MBMS: This author carried out the study and wrote the main part of the paper.

ThJTC and GVDW: These authors participated in the design of the study and commented the paper.

## Pre-publication history

The pre-publication history for this paper can be accessed here:


